# Learning from the past: a scoping review of hospital disaster preparedness assessment

**DOI:** 10.1186/s12873-026-01474-2

**Published:** 2026-01-09

**Authors:** Prinka Singh, Sujan Sapkota, Nebil Achour, Luca Ragazzoni, Hamdi Lamine

**Affiliations:** 1 CRIMEDIM-Center for Research and Training in Disaster Medicine, Humanitarian Aid and Global Health, Università Del Piemonte Orientale, Novara, 28100 Italy; 2 Department for Sustainable Development and Ecological Transition, Università Del Piemonte Orientale, Vercelli, 13100 Italy; 3 HERD International, Sainbu Awas Cr-10 Marga, Bhaisepati, Lalitpur, Nepal; 4 School of Allied Health and Social Care, Faculty of Health, Education, Medicine and Social Care, Anglia Ruskin University, East Road, Cambridge, CB1 1PT UK; 5 Department of Community Health Nursing, College of Nursing, University of Hail, Hail, Saudi Arabia

**Keywords:** Emergency planning, Hospital disaster preparedness, Hospital resilience, Preparedness gaps, Scoping review

## Abstract

**Background:**

Despite the recognized importance of hospital disaster preparedness (HDP), efforts to improve it have been limited. Improvement in HDP will need an in-depth comprehension of the prevailing gaps in addition to evidence of the best practices. However, a comprehensive review that synthesizes the findings from HDP assessments across diverse contexts and translates them into actionable insights is currently lacking. Considering these gaps, this scoping review aims to examine current practices, identify recurring gaps, consolidate best practices, and provide actionable recommendations through an in-depth literature review.

**Methods:**

A scoping review was done using the Preferred Reporting Items for Systematic Reviews and Meta-Analysis Extension for Scoping Reviews (PRISMA-ScR) guidelines. A comprehensive search strategy using keywords related to HDP was carried out in three databases: PubMed, Scopus, and Web of Science. Eligibility criteria were defined according to the population, intervention, comparison, and outcome. The search included studies from January 2015 to December 2024. Screening and data extraction were done by two independent reviewers, and the extracted data were subjected to a narrative synthesis. Data extraction and analysis were manually performed using Excel.

**Results:**

46 eligible articles were identified from 10,656 records, the majority from Iran and Saudi Arabia. Cross-sectional studies dominated, and the majority utilized the hospital safety index tool. Two-thirds of hospitals reported a moderate level of preparedness. Substantial variability in hospital safety scores was observed, with structural safety ranging from 28% to 76.16%, nonstructural safety from 17.02% to 73.2%, and functional preparedness from 11.35% to 95%. Most hospitals lacked adequate structural safety, backup communication systems, proper safety measures for furniture and medical equipment, training programs, comprehensive emergency planning, staff welfare strategies, and adequate logistics and supplies.

**Conclusions:**

HDP should be viewed as an evolving, ongoing process, requiring a balanced HDP framework that addresses all aspects of preparedness, region-specific guidelines tailored to the unique needs and risks of the hospital, and context-driven interventions to enhance hospital resilience.

**Supplementary Information:**

The online version contains supplementary material available at 10.1186/s12873-026-01474-2.

## Background

Disasters are becoming increasingly frequent and severe, driven by global trends such as climate change, rapid urbanization, and political instability [1–6]. Earthquakes, floods, pandemics, industrial accidents, and armed conflicts continue to disrupt societies, overburden emergency systems, and threaten public health infrastructure worldwide [1, 3]. Within this escalating risk environment, hospitals play a central role in disaster response, not only by delivering urgent medical care, but also by maintaining essential services, coordinating community-level interventions, and often functioning as command centers during large-scale emergencies [3, 4, 6–9].

When disasters strike, hospitals are expected to function under extreme pressure, often with compromised infrastructure, limited resources, and surges in patient demand [10–13]. Their operational capacity in such situations determines not only health outcomes but also public trust and societal resilience [14]. In this context, hospital disaster preparedness (HDP), defined as a hospital’s capacity to plan for, respond to, and recover from emergencies while sustaining critical functions, has emerged as a global public health priority [9, 15, 16].

To support and strengthen health system resilience, organizations such as the World Health Organization (WHO) and Pan American Health Organization, along with national agencies, have developed structured tools and checklists to assess HDP [7, 11, 17–19]. These instruments assess multiple dimensions of preparedness, including structural and nonstructural safety, emergency operation plan, human resources, surge capacity, logistics, communication systems, coordination mechanisms, and continuity of care [7, 11, 17–19]. However, the effectiveness of these efforts largely depends on how systematically preparedness is assessed, monitored, and translated into actionable improvements at the health facility level [15, 20].

Despite the growing interest in HDP, the existing body of literature remains fragmented. Studies vary widely in the frameworks and indicators they use, the geographic and institutional settings they examine, and the depth of their assessments [21, 22]. While some focus on emergency planning and leadership structures, others prioritize logistical readiness or infrastructure safety [21, 22]. As a result, there is no unified understanding of how hospitals are performing globally across key domains of preparedness. Moreover, the translation of assessment findings into concrete policy actions or hospital-level improvements is seldom addressed, highlighting a persistent gap between evaluation and implementation.

Although previous reviews have explored HDP, they are often limited in scope, focusing on single preparedness domains, a specific country or region, or assessment tools [21–24]. To date, no review has comprehensively synthesized evidence across structural, nonstructural, and emergency and disaster management domains while critically analyzing common gaps and their implications for hospital resilience. As a result, decision-makers and hospital managers are left without a cohesive understanding of existing strengths, recurring gaps, or transferable best practices, thereby hindering efforts to formulate targeted and effective preparedness strategies. Thus, to bridge this gap and facilitate effective preparedness strategies and interventions for strengthening hospital resilience against future disasters, this study aims to examine current practices, pinpoint recurring gaps, consolidate best practices, and provide actionable recommendations through a comprehensive examination of existing literature on the assessment of HDP across the multidimensional spectrum of structural, nonstructural, and emergency and disaster management aspects. It is important to note that these results are not representative of the HDP levels but rather reflect only what has been published in the existing literature. Nevertheless, it can provide valuable insight into the existing gaps and offer recommendations for planners and policymakers.

## Methods

### Study design, search strategy, and selection criteria

This scoping review was conducted following the Preferred Reporting Items for Systematic Reviews and Meta-Analyses extension for Scoping Reviews (PRISMA-ScR) [25]. The process was further guided by Arksey and O’Malley’s five-stage framework for scoping reviews, which involves: (i) defining the research question; (ii) identifying relevant studies; (iii) selecting studies; (iv) extracting and charting data; and (v) collating, summarizing, and presenting the findings [26].

Articles were selected from PubMed, Scopus, and Web of Science databases. The search strategy involved a string of keywords related to HDP, including terms such as hospital, health facilities, healthcare facilities, disaster preparedness, emergency preparedness, hospital preparedness, hospital resilience, hospital safety index, structural preparedness, structural safety, nonstructural preparedness, and nonstructural safety. Keywords were connected using Boolean operators “OR” and “AND” (Appendix A).

The study eligibility criteria were formulated based on the population, intervention, comparison, and outcome framework [27], see Table 1.

**Table 1:** Inclusion and exclusion criteria

	Inclusion criteria	Exclusion criteria
Population	• Studies focusing on hospitals	• Studies related to other healthcare facilities such as primary healthcare centers or local clinics • Studies concentrating on singular hospital departments (e.g., pharmacy, neonatal intensive care unit, pediatric departments)
Intervention	• Assessment of HDP	• Assessment of routine activities
Comparison	None	
Outcome	• Studies that assess any of the following elements of HDP: • Structural element • Nonstructural element • Emergency and disaster management element	• Studies that evaluate the knowledge, attitude, practice, perception, or perspective of health workers on HDP • Studies focusing solely on one specific disaster (e.g., floods, road traffic accidents, coronavirus disease of 2019, etc.)
Study type	• Peer-reviewed quantitative studies written in English	• Qualitative studies, reviews, books, editorials, letters to editors, commentaries, conference abstracts, and perspectives • Publications that were not peer-reviewed • Grey literature • Articles in a language other than English
Timeline	Studies published between the start of 2015 and the end of 2024	
Settings	Regardless of any country and geography	

Abbreviation: HDP, hospital disaster preparedness

Note: Qualitative studies were excluded to maintain comparability across studies. Although qualitative research offers valuable contextual insights, including it would have introduced heterogeneity in outcome measures, limiting the ability to synthesize evidence

### Study selection and data collection

After eliminating duplicates, two independent reviewers (PS and SS), first screened the titles and abstracts of the identified articles. Then they independently evaluated the full-text articles against the specified inclusion and exclusion criteria. Any discrepancies that arose were resolved through discussion between the reviewers at each stage of the screening process.

Subsequently, both reviewers (PS and SS) independently extracted relevant data from the eligible full-text articles using a predefined template. Since the hospital safety index (HSI) tool is a comprehensive instrument that covers all aspects of HDP more effectively than other tools, the HSI tool 2015 version was used as a reference for data extraction purposes [28, 29]. Data on study characteristics, such as title, year, study design, country, sample size, and findings and recommendations related to hospital structural, nonstructural, and emergency and disaster management components were extracted. Data extraction was manually performed using Microsoft Excel spreadsheet version 2401.

Structural components encompass the load-bearing elements of a building, such as columns, beams, walls, floor slabs, and foundations. On the other hand, nonstructural components include elements such as architectural elements, emergency access and exit routes in hospitals, critical systems (such as electricity, water supply, waste management, and fire protection), and safety of equipment like medical, laboratory, and office equipment. The emergency and disaster management aspect evaluates a hospital’s organizational and personnel preparedness, along with its essential operations, to deliver patient services during emergencies or disasters [28].

### Data synthesis and analysis

A narrative synthesis was carried out to integrate the findings, conducted by two reviewers (PS and SS). The synthesis was guided by the Popay et al. (2006) framework, which provides a structured method for summarizing and explaining findings across heterogeneous studies. This involved [30]:

1. **Developing a preliminary synthesis**: Key characteristics, preparedness domains, assessment tools, and findings were extracted and tabulated.
2. **Exploring relationships within and between studies**: Patterns, recurring gaps, and differences across structural, nonstructural, and emergency and disaster management domains were identified.
3. **Assessing the robustness of the synthesis**: The frequency of reported gaps, consistency of findings across geographic and institutional contexts, and quality of evidence were considered to support interpretation and conclusions.

Data synthesis and analysis were manually performed using Microsoft Excel spreadsheet version 2401.

## Results

### Study selection

**Fig. 1:**
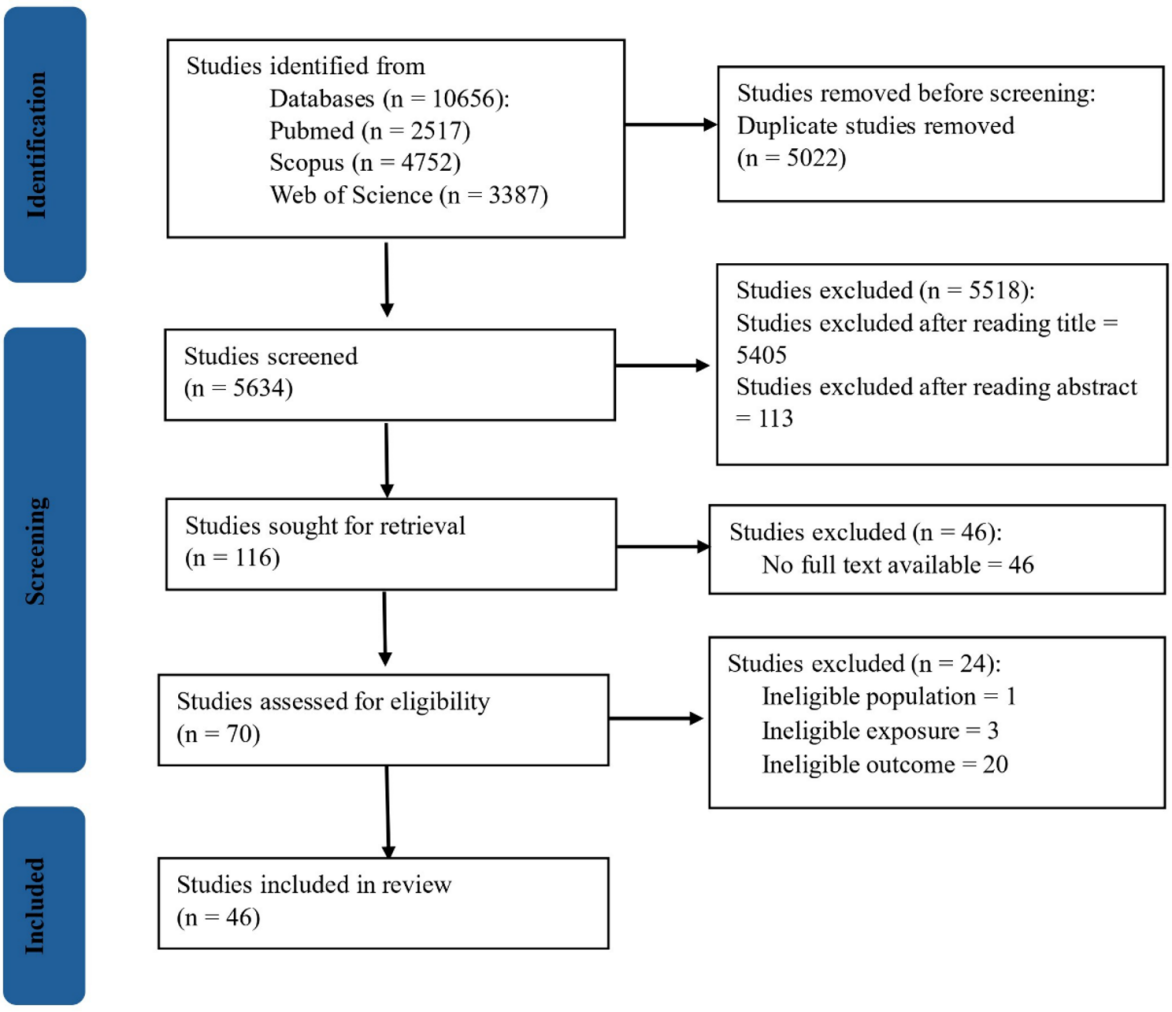
PRISMA flow diagram

The initial database search yielded a total of 10,656 records. After removing duplicates and screening titles and abstracts, 116 studies were sought for retrieval, of which 70 full-text studies were thoroughly reviewed. Of these, 46 studies met the inclusion criteria and were included in the final synthesis. The PRISMA flow diagram illustrating the study selection process is presented in Fig. 1.

### Quality assessment

All included studies underwent a structured quality appraisal process. Quality assessment of cross-sectional studies utilized the “Strengthening the Reporting of Observational Studies in Epidemiology” checklist for cross-sectional studies, which comprises 22 components [31]. The highest score obtained from this checklist was 30, with a minimum acceptable rating set at 15 [32]. In the case of quasi-experimental studies, the National Institutes of Health quality assessment tools for before-after (pre-post) studies with no control group were employed [33]. This checklist consisted of 12 questions that assessed various criteria within the articles, leading to categorization into good, fair, or poor quality [33]. Studies categorized as poor quality were excluded from the final inclusion list [34]. Studies that did not conform to these specified designs (such as mixed-method studies, longitudinal evaluation studies, and comparative studies) were not subjected to assessment. The evaluation process involved two independent reviewers (PS and SS) who assessed the quality of the included studies, resolving any discrepancies through discussion with each other. All studies met the minimum quality standards, and therefore, none were excluded based on quality concerns.

### Study characteristics and context

**Fig. 2:**
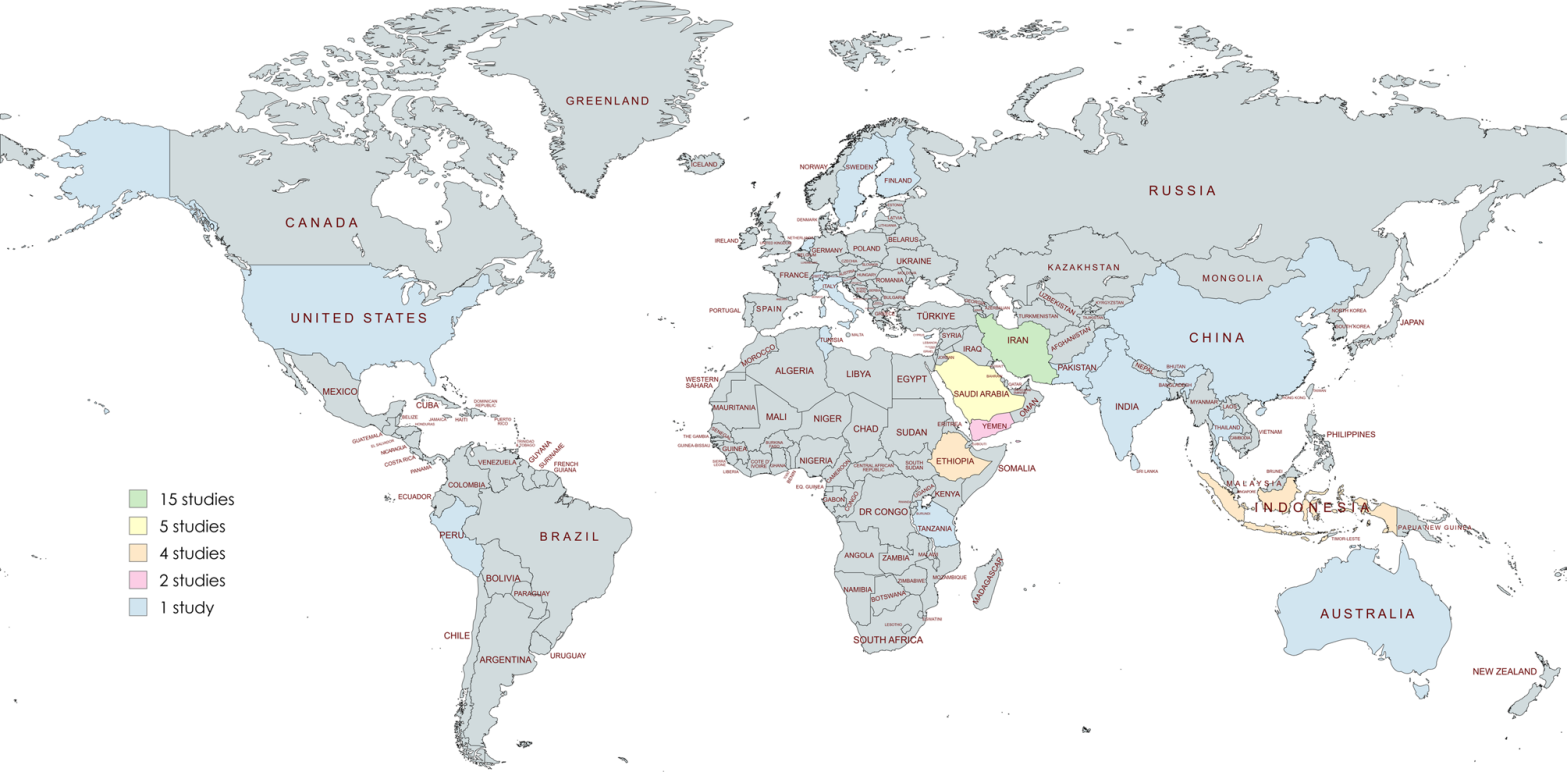
Map showing country-wise study distribution

A total of 46 studies were included in the review, encompassing research conducted across 21 countries. The majority of studies originated from low and middle income countries (LMICs), reflecting the growing attention toward HDP in resource-constrained settings. Iran accounted for the highest number of studies (*n* = 15, 32.61%), followed by Saudi Arabia (*n* = 5, 10.87%) (Fig. 2).

**Fig. 3:**
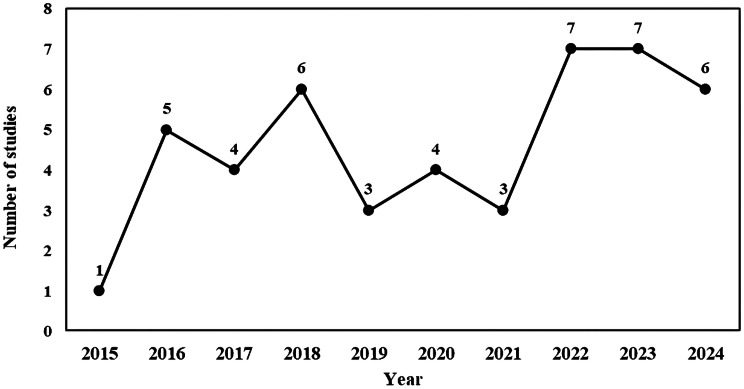
Line chart showing year-wise study distribution

The number of studies on HDP assessment has shown a fluctuating but overall upward trend from 2015 to 2024. The highest number was observed in 2022 and 2023, with 7 studies each year (Fig. 3).

The predominant study types were cross-sectional (*n* = 35, 76.09%), with a smaller representation of quasi-experimental, mixed method studies, longitudinal evaluation studies, and comparative studies. A wide range of assessment tools were employed, with standardized WHO tools predominating. HSI was the most frequently used tool (*n* = 19, 41.31%) [28], with 6 studies reporting contextual adaptations. The WHO hospital emergency response checklist was used in 12 studies (26.09%) [35], with 3 of them modifying the tool to meet context-specific needs. Additionally, 11 studies (23.91%) used tools developed by the researchers themselves, while few studies (*n* = 3, 6.52%) adopted a hybrid approach by combining two standardized tools, most commonly the WHO national health sector emergency preparedness and response tool and the WHO hospital emergency response checklist [35, 36]. Nearly all studies (*n* = 44, 95.65%) evaluated the disaster and emergency management preparedness of hospitals, while 14 (30.43%) and 24 (52.17%) studies assessed the structural and nonstructural components of hospitals respectively (Table 2).

**Table 2:** Study characteristics and context

S.*N*	Reference	Year of Publication	Country of origin	Sample size	Study design	Tool used	HDP component assessed
1	[1]	2024	Iran	6	Cross-sectional	Modified WHO hospital emergency response checklist	FS
2	[2]	2024	Iran	1	Quasi-experimental	Modified HSI 2015 version	NS
3	[37]	2024	Indonesia	5	Mixed	HSI 2015 version	FS, NS, SS
4	[3]	2024	Iran	23	Longitudinal	Modified HSI 2015 version	FS, NS, SS
5	[38]	2024	Finland	28	Cross-sectional	WHO hospital emergency response checklist	FS
6	[4]	2024	Iran	55	Cross-sectional	Modified HSI 2015 version	FS, NS, SS
7	[15]	2023	Saudi Arabia	63	Cross-sectional	WHO national health sector emergency preparedness and response tool and WHO hospital emergency response checklist	FS, NS
8	[6]	2023	Ethiopia	17	Cross-sectional	WHO hospital emergency response checklist	FS
9	[17]	2023	Netherlands	20	Cross-sectional	WHO hospital emergency response checklist	FS
10	[39]	2023	Pakistan	5	Cross-sectional	HSI 2015 version	FS, NS, SS
11	[40]	2023	Peru	18	Cross-sectional	HSI first version	FS, NS, SS
12	[5]	2023	Ethiopia	10	Cross-sectional	Researcher developed tool	FS
13	[7]	2023	Iran	604	Cross-sectional	Modified HSI tool	FS, NS, SS
14	[8]	2022	Australia	6	Cross-sectional	HSI 2015 version	FS, NS, SS
15	[20]	2022	Ethiopia	4	Mixed	HSI 2015 version	FS
16	[10]	2022	Iran	1	Quasi-experimental	Researcher developed tool	FS
17	[11]	2022	Indonesia	9	Cross-sectional	HSI 2015 version	FS, NS, SS
18	[41]	2022	Tunisia	9	Cross-sectional	HSI 2015 version	FS, NS, SS
19	[42]	2022	Sweden	39	Cross-sectional	Researcher developed tool	FS, NS
20	[43]	2022	Ethiopia	10	Cross-sectional	HSI 2015 version	FS
21	[44]	2021	Saudi Arabia	63	Cross-sectional	WHO national health sector emergency preparedness and response tool and WHO hospital emergency response checklist	FS, NS
22	[16]	2021	Saudi Arabia	4	Quasi-experimental	WHO hospital emergency response checklist	FS
23	[18]	2021	Thailand	11	Cross-sectional	Researcher developed tool	FS
24	[45]	2020	Indonesia	5	Cross-sectional	Modified HSI 2015 version	FS
25	[46]	2020	Lebanon	24	Cross-sectional	Researcher developed tool	FS, NS
26	[47]	2020	Iran	1	Cross-sectional	WHO hospital emergency response checklist	FS
27	[19]	2020	Indonesia	15	Cross-sectional	HSI 2015 version	FS, NS, SS
28	[48]	2019	Iran	15	Cross-sectional	Modified WHO hospital emergency response checklist	FS
29	[49]	2019	Iran	1	Quasi-experimental	Modified WHO hospital emergency response checklist	FS
30	[50]	2019	Sri Lanka	1	Mixed	Researcher developed tool	FS, NS
31	[51]	2018	Iran	1	Quasi-experimental	HSI first version	FS, NS, SS
32	[52]	2018	Tanzania	25	Cross-sectional	WHO national health sector emergency preparedness and response tool	FS, NS
33	[53]	2018	USA	80	Cross-sectional	Researcher developed tool	FS
34	[54]	2018	Switzerland	83	Cross-sectional	Researcher developed tool	FS
35	[12]	2018	Yemen	10	Cross-sectional	WHO hospital emergency response checklist	FS, NS
36	[55]	2018	Iran	18	Cross-sectional	WHO hospital emergency response checklist	FS
37	[56]	2017	Saudi Arabia	13	Cross-sectional	WHO toolkit for assessing health-system capacity for crisis management and WHO hospital emergency response checklist	FS
38	[57]	2017	Iran	6	Cross-sectional	HSI 2015 version	FS, NS, SS
39	[58]	2017	Iran	8	Cross-sectional	HSI first version	NS
40	[13]	2017	India	190	Mixed	Researcher developed tool	FS
41	[9]	2016	Italy	15	Cross-sectional	WHO hospital emergency response checklist	FS
42	[59]	2016	Iran	421	Cross-sectional	Modified HSI first version	FS, NS, SS
43	[60]	2016	Saudi Arabia	14	Cross-sectional	Researcher developed tool	FS, NS
44	[61]	2016	China	65	Cross-sectional	Researcher developed tool	FS
45	[62]	2016	Iran	2	Cross-sectional	HSI first version	FS, NS, SS
46	[63]	2015	Yemen	9	Comparative	WHO hospital emergency response checklist	FS

Abbreviation: FS, Functional Safety; HSI, Hospital Safety Index; NS, Nonstructural Safety; SS, Structural Safety; WHO, World Health Organization

### Key findings

#### Overall preparedness

A total of 2,033 hospitals were examined across the 46 included studies. Of these, 25 studies encompassing 1,461 hospitals reported categorized data on HDP levels. Among them, 146 (10%) were classified as having high preparedness, 973 (66.6%) had moderate preparedness, and 342 (23.4%) were reported to have low preparedness levels.

Higher levels of preparedness were generally observed in hospitals located in high-income countries such as Finland and Australia. In contrast, hospitals in LMICs demonstrated moderate to low preparedness levels. However, exceptions were noted. For example, a few studies from Iran and Indonesia reported high levels of HDP, indicating variability even within the same national context. The review also revealed significant disparities in preparedness among different types of hospitals within the same national setting. For instance, in Pakistan and Iran, military and private hospitals were generally better prepared compared to public and community hospitals, highlighting intra-country inequities in disaster readiness. The detailed data extraction sheet is provided in Appendix B.

### Findings on structural safety

The review found considerable differences in hospital structural safety scores, ranging from as low as 28% to as high as 76.16%. A contributing factor to these differences was infrastructure vulnerability as reported in five studies [11, 19, 41, 57, 62]. Aging buildings, poor structural conditions, and the use of substandard construction materials were common challenges. Moreover, many of these facilities were situated in hazard-prone areas, such as flood zones and steep terrain, yet lacked adequate, context-specific measures to reduce their exposure to disaster risks. Conversely, hospitals with higher structural safety scores generally complied with established architectural and engineering standards, used quality construction materials, and incorporated universal design principles. These facilities further benefited from timely retrofitting and post-disaster reconstruction efforts, which enhanced their resilience.

### Findings on nonstructural safety

Similar to structural safety, the nonstructural component exhibited notable variability, with mean safety scores ranging from as low as 17.02% to as high as 73.2%, reflecting uneven preparedness across hospitals.

One of the most frequently cited weaknesses was the inadequate securing of office and storeroom furniture, medical equipment, and shelving. This issue was highlighted in 8 studies, with preparedness scores in this domain ranging from 0% to 56%. Equally concerning was the absence or limited availability of backup communication systems, also noted in 8 studies. While most hospitals had installed backup generators, few had established alternative communication options such as walkie-talkies, radio systems, or satellite phones. In contrast, areas such as waste management systems, water supply systems, and architectural elements generally showed higher levels of preparedness, with some studies reporting maximum safety scores reaching 100%. Table 3 presents the summary of gaps identified in nonstructural safety across the reviewed studies.

**Table 3:** Summary of identified gaps in nonstructural safety

Gap	Number of studies	Reference
Absence or limited availability of backup systems	8	[12, 41, 42, 44, 46, 50, 52, 58]
Furniture, medical devices, and shelving not anchored or braced	8	[2, 4, 7, 11, 19, 57, 59, 62]
Lack of fencing and access control measures; obstructed emergency routes	4	[15, 41, 44, 52]

### Findings on emergency and disaster management

Functional preparedness emerged as the most extensively reported domain across the reviewed studies. While some areas, such as command and control, triage, and surge capacity, exhibited signs of moderate preparedness, others, particularly disaster planning, training, and post-disaster recovery, were markedly deficient. Preparedness scores across studies varied widely, ranging from 11.35% to 95%. Table 4 presents multiple gaps identified across the various subdomains within the functional component (Table 4).

**Table 4:** Summary of identified gaps in functional safety

Domain	Gap	Number of studies	Reference
Command and control	Disaster committees exist in theory but lack activation, coordination, and practical implementation	6	[5, 6, 43, 45, 52, 60]
	Key personnel lack training and clarity on their roles during disasters	5	[6, 20, 41, 43, 44]
	Missing or insufficient emergency operations center; absence of alternate command locations	3	[15, 43, 44]
Disaster planning	No comprehensive plans; infrequent review and update of plan	16	[5, 12, 15, 20], 40– [44, 52, 54, 56], 60– [63]
Training	Lack of regular, comprehensive, and evaluated training; unannounced drills or simulations rarely conducted	14	[6, 15, 20], 41– [44, 46, 50, 52, 54, 56, 60, 63]
Human resource	Lack of systems to recall off-duty staff or mobilize staff from other facilities	8	[5, 15, 20, 46, 49, 54, 55, 60]
	Contact databases not regularly updated; missing key information	5	[20], 42– [44, 52]
	Inadequate psychological support, shift management, incentives, or disaster-specific training	4	[6, 20, 48, 60]
Logistics and supply chain	Shortage of medical supplies, ambulances, ventilators, and other essential equipment	11	[5, 15, 20, 43, 44, 46, 50], 52– [54, 61]
	Stores not maintained; inventories outdated or missing critical supplies	10	[5, 6, 42, 46, 50, 52, 54], 60– [62]
	No formal vendor agreements for emergency	3	[20, 43, 44]
	No emergency fund allocation or budget mechanism	3	[20, 41, 63]
Safety and security	No evacuation plans; absence of designated decontamination areas; no alternative patient routes	12	[6, 8, 15, 20, 41, 43, 44, 46, 48, 52, 54, 60]
	Inadequate personnel identification mechanism; poor access control; lack of military coordination; insecure data storage	6	[6, 13, 20, 41, 48, 54]
Triage	No designated areas for triage or alternative sites; no clear identification of entrance and exit routes to the triage	7	[6, 12, 20, 44, 46, 48, 52]
	Absence of standardized triage methods; CBRNE protocols not in use	5	[6, 42, 48, 52, 60]
Surge capacity Surge capacity	Inadequate comprehensive surge capacity planning; lack of strategies to manage resources during patient surge	9	[6, 15, 20, 42, 44, 48, 52, 53, 60]
	Insufficient designated care areas and contingency sites for patient overflow	8	[6, 15, 44, 46, 48, 52, 53, 60]
Post-disaster recovery	No designated teams or documented post-disaster recovery plans	5	[6, 46, 48, 49, 60]
	Absence or inadequate recovery assistance programs such as counseling, debriefing, and recognition for staff and volunteers	4	[15, 44, 48, 60]

Abbreviations: CBRNE, Chemical, Biological, Radiological, Nuclear, and Explosive Materials

### Targeted interventions in enhancing HDP

Among the studies included in the review, only five designed and implemented specific interventions aimed at enhancing HDP [2, 10, 16, 49, 51]. Table 5 illustrates the scope of these interventions, along with their corresponding improvements expressed as percentage increased in preparedness scores. The percentage increase represents the measured improvement in safety or preparedness performance compared to the pre-intervention baseline.

**Table 5:** Impact of interventions on HDP across key components

Domain	Intervention implemented	Percentage increased	Reference
Building integrity	Renovation of hospital building	NA	[51]
Architectural safety	Installation of guards and fences on walls and rooftops; addition of stairs and stair railings	10.53%	[2]
Water supply	Locking tanker entry doors; restricted access to storage areas; installation of backup and supplementary pumps	33.32%	[2]
Medical gas system	Relocation of oxygen production and storage areas; integration of alternative sources	50%	[2]
Heating, Ventilation, and Air Conditioning	Installation of clamping pipe connections; bracing cables on equipment; use of flexible connectors	12%	[2]
Office and storeroom furnishing	Installation of braces, secure cupboards, screw fasteners, and strapping of cages and computers	50%	[2]
Medical and laboratory equipment	Installation of a central sterile services department; modernization of equipment	10.50%	[2]
Command and control	Training, assignment, and reassignment of focal and alternate persons; emergency operation center equipped with computers, internet, and contact lists; personnel recall system	12.96% to 87.5%	[10, 16, 49, 51]
Surge capacity	Surge capacity planning and testing; cancellation of elective services; expansion of space; partnerships with gyms and emergency medical service for space expansion and transport support	27.27% to 109.09%	[10, 16, 45]
Continuity of essential service	Updated contact for emergency fuel supplier and repair company; dual-fuel energy systems; fire detection systems	7.46% to 70.59%	[10, 16, 45]
Safety and security	Horizontal and vertical evacuation plans; restart plans for critical units such as laboratory, radiology department, and operating room; signage; rerouting plans; crowd and parking control plan; gate control plan; partnership with police	18.49% to 78.57%	[10, 16, 45]
Communication	Telephone call recording system; trained spokesperson assignment; designated space for media/community interaction; text message communication; updated contact list of external stakeholders; patient tracking system	16.13% to 128%	[10, 16, 45]
Triage	Appointment of experienced triage staff; triage training; designated triage areas and routes	14.29% to 65%	[10, 16, 45]
Human resource	Training needs assessment; support plans for staff families; volunteer role definition; temporary liability insurance; updated staff contact list	58.70% to 88.89%	[10, 16, 45]
Logistics and supply management	Agreements with pharmaceutical companies for emergency supply	18.37% to 119.18%	[10, 16, 45]
Plan	Development of a comprehensive all-hazard disaster plan, covering mitigation, preparedness, and response	NA	[10, 16, 45]
Training	Disaster response training for staff; biannual simulation drills addressing all potential local hazards	NA	[10, 16]
Post-disaster recovery	Development of post-disaster recovery plans; multidisciplinary psychological support; mental health support for staff and patients; post-disaster volunteer role descriptions	21.21% to 1400%	[10, 16, 45]

Abbreviations: NA, not available

The most commonly used framework guiding these interventions was the FOCUS-PDCA (Find, Organize, Clarify, Understand, Select-Plan, Do, Check, Act) quality improvement model, employed in two studies to guide systematic cycles of planning, implementation, evaluation, and refinement [2, 10]. Additionally, one study employed focus group discussions with multidisciplinary hospital staff to identify preparedness gaps and co-develop contextually tailored solutions [51].

### Recommendations to enhance HDP

As per the studies included in the review, the top five key recommendations suggested to strengthen HDP for future resilience were as follows:

#### Prioritize continuous staff education and emergency drills

Over half of the studies (*n* = 25, 54.35%) emphasized the importance of regular staff training, and 14 studies reported that comprehensive, evaluated training programs and unannounced drills were rarely conducted. Hospitals should therefore implement ongoing education programs, simulation-based exercises, and mandatory annual drills, coupled with evaluation mechanisms, to ensure that healthcare personnel remain competent, confident, and responsive during disasters.

#### Develop and regularly update comprehensive disaster plans

Nearly half of the studies (*n* = 21, 45.65%) underscored the importance of formulating comprehensive, all-hazard disaster preparedness plans that are periodically reviewed. Specifically, 16 studies reported that hospitals lacked comprehensive plans and conducted infrequent reviews or updates. Thus, hospitals should formulate and regularly update all-hazard plans covering emergency response, surge capacity management, resource allocation, and post-disaster recovery. Ensuring these plans are actionable and periodically reviewed will enhance organizational readiness and resilience.

#### Secure nonstructural components to prevent injuries and disruption

A significant subset of studies (*n* = 7, 15.22%) reported that unsecured nonstructural elements, such as medical equipment, furniture, and mobile units, contribute to injuries and operational disruptions during disasters. Additionally, 8 studies specifically noted that furniture, medical devices, and shelving were not anchored or braced, increasing the risk of harm and service interruption. Hospitals should thus anchor or stabilize nonstructural elements and maintain accessibility of emergency equipment to safeguard staff, patients, and continuity of services.

#### Foster interagency coordination and community engagement

Around 13.04% of studies (*n* = 6) emphasized enhanced coordination with external stakeholders, including ministries, pre-hospital services, neighboring hospitals, non-governmental organization, schools, communities, and security forces. Hospitals should establish formal mechanisms for interagency coordination and community engagement, ensuring an integrated, well-resourced, and timely emergency response. Such mechanisms help address human resource and logistics gaps, reported in 8 and 11 studies respectively, by facilitating the sharing of personnel, equipment, and other essential resources during disasters, thereby enhancing overall preparedness and response capacity.

#### Retrofit or reconstruct vulnerable infrastructure

Structural preparedness was consistently reported as low across multiple studies (*n* = 5, 10.87%), placing hospitals at high risk during disasters. Therefore, hospitals with outdated buildings should prioritize retrofitting or reconstruction to meet current seismic and hazard-resilient standards, particularly in resource-constrained settings, to reduce the risk of collapse, injuries, and service interruption.

#### Establish dedicated budgets for preparedness activities

Five studies (10.87%) highlighted that limited financial resources constrained the implementation of disaster preparedness measures. Hospitals should allocate dedicated budgets covering staff training, disaster planning, emergency resource procurement, and infrastructure improvements.

## Discussion

Given the increasing frequency and severity of disasters globally, evaluating the preparedness of healthcare facilities has become more crucial than ever [6, 10]. This review is the first to systematically synthesize global evidence on HDP across structural, nonstructural, and functional domains. In contrast to earlier reviews that focused on a single domain or were limited to specific countries or regions, this study offers a broad, cross-cutting perspective, revealing consistent patterns and enduring gaps in HDP across the globe.

The present review uncovers a deeply troubling picture of HDP worldwide. Only a minority of hospitals (10%) reported a highly prepared level, while two-thirds scored merely moderate, and nearly a quarter remained low-prepared. Such figures imply that far fewer than one in ten hospitals would be deemed highly prepared. Importantly, moderate preparedness, as reported in most studies, should not be interpreted as an adequate standard. It often reflects partial readiness, such as having plans or committees in place, without sufficient capacity to ensure patient and staff safety, maintain continuity of care, or sustain functionality during disasters [28]. From a risk perspective, hospitals at this level remain vulnerable, and require short-term interventions to reduce risks to patients, healthcare workers, and essential services and to maintain functionality during and after emergencies [28]. This is consistent with results from other reviews as well [21, 24]. For example, systematic assessments in Iran found that hospitals were only about half-prepared on average, with an overall preparedness score of 53% [21]. Similarly, another review covering sub-Saharan Africa (SSA) concludes that hospitals in SSA are not adequately prepared for possible disaster strikes and emergencies due to prevailing health system challenges [24]. Thus, these findings suggest that despite growing attention, global progress in HDP has been modest and has not translated into widespread disaster resilience.

The present review also identified a marked increase in HDP research over the past decade. This surge can be attributed to several factors, including the impact of multiple disasters and events. For instance, between 2012 and 2017, the WHO recorded over 1200 outbreaks in 168 countries, involving both new and re-emerging infectious diseases [43]. Additionally, the occurrence of disasters such as hurricanes, floods, earthquakes, fires, droughts, terrorist attacks, volcanic eruptions, and chemical accidents has shown an increasing pattern [8, 52, 57, 62]. Furthermore, the coronavirus disease of 2019 pandemic, which began in late 2019 and escalated in 2020 [64], could have influenced research priorities, leading to a rise in studies focused on HDP, pandemic preparedness, and overall healthcare system resilience. Yet despite this surge in research, low to moderate preparedness levels persist, highlighting a disconnect between assessment and implementation. Much of the literature documents preparedness gaps, but comparatively little demonstrate how these weaknesses are addressed or mitigated over time, limiting the practical impact of this growing body of research.

The geographic distribution of included studies reveals a notable concentration of research from Iran, which accounted for nearly one-third of all studies in this review. Several factors may explain this observation. Iran is a disaster-prone country, frequently affected by earthquakes, floods, and other hazards, which has heightened national attention on disaster risk reduction within the healthcare sector [2, 4, 48]. Additionally, the widespread use of standardized assessment tools, such as the HSI, combined with a strong interest in disaster medicine, may have contributed to the higher number of studies on HDP [3, 7, 59]. Nonetheless, this concentration limits the global generalizability of findings. While challenges identified in Iranian hospitals are relevant to other LMICs, differences in governance, health system organization, resources, and hazard profiles may restrict applicability to high-income or fragile settings. As a result, there is the need for more evidence from underrepresented regions to achieve a globally representative understanding of HDP.

Another key finding of the review was that private and military hospitals demonstrated higher levels of HDP compared to public hospitals, raising important equity concerns.

Public hospitals are the primary providers of free or low-cost health services and are heavily relied upon during disasters [65]. Lower preparedness in these facilities may therefore lead to service disruptions that disproportionately affect populations dependent on public sector care in emergencies. In contrast, private and military hospitals often benefit from more stable financing, stronger governance, and better access to trained personnel and emergency resources [65]. This imbalance highlights an unequal distribution of disaster risk within health systems and emphasizes the need for equity-oriented preparedness policies, targeted investment in public hospitals, and the incorporation of equity-sensitive indicators in HDP assessments.

Another salient finding was the overwhelming focus on functional preparedness. Most studies in the current review assessed emergency plans, drills, disaster committees, and training, but far fewer evaluated the safety of buildings, equipment, or lifelines. This disparity likely arises from the relative simplicity of evaluating managerial and procedural components compared to structural and nonstructural integrity, which requires technical expertise, resources, and access to engineering assessments. Furthermore, functional preparedness is more immediately modifiable, making it an attractive focus for both researchers and hospital administrators. However, this bias is disturbing because real-world disasters frequently inflict damage on both buildings and infrastructure [66]. The WHO’s Safe Hospitals initiative and the Sendai Framework make this clear that health facilities must remain accessible and functional, at maximum capacity, immediately after a disaster strikes [67, 68]. However, the present findings suggest that global research lags behind these policy goals. Most studies intensely evaluate management capacity while often overlooking whether the hospital can physically withstand a disaster. These findings align with the critique by Munasingh et al., who noted that most HDP studies fail to adopt a truly holistic framework [22]. Yet, structural integrity and utility resilience are foundational, without them, even the best emergency plans are rendered ineffective. This review therefore reinforces the call for a multidimensional preparedness model that integrates all domains under a unified framework.

Moreover, the wide variation in preparedness scores across the reviewed studies reflects considerable heterogeneity in HDP. This variability seems to result from both contextual differences and methodological inconsistencies in assessment approaches. Factors such as national income, health system governance, and prior disaster experience may affect preparedness levels, as seen in generally higher scores in high-income countries [8, 38]. Additionally, differences in assessment tools, scoring frameworks, and classification criteria likely contribute to this heterogeneity. While these variations highlight significant inequities in HDP, they also limit the direct comparability of preparedness scores across studies.

The review documented the proliferation of instruments where the modified WHO standardized tool was widely used, along with researcher-developed questionnaires and checklists. Similar findings were observed in other review [69, 70]. For instance, Husaini et al. cataloged this variety, noting that most of the studies used modified tools by experts, with several relying on abbreviated WHO HSI versions [69]. This diversity reflects a lack of a standardized approach and no consensus about what preparedness encompasses and what elements need to be present in a preparedness evaluation tool [24, 70]. In practice, this means two consequences: first, without a core set of tested indicators, research outcomes lack comparisons across settings and over time [70]. Second, many studies used researcher-designed surveys or non-validated checklists, which raises doubts about measurement quality [70]. Therefore, developing a modular system consisting of fixed, must-have criteria as well as optional criteria is recommended [70]. That approach would provide minimum standards and comparability as well as support individualization by adding variables depending on the contexts [70].

The review also observed methodological weaknesses in literature. Indeed, the bulk of the literature consists of descriptive cross-sectional surveys rather than experimental or longitudinal studies. In other words, very few publications have evaluated whether training programs or new protocols improve readiness, which is a critical implementation gap. Therefore, the HDP evidence base is rich in description but largely fails to demonstrate what works in practice. For example, Farah et al. note that in SSA the published literature remains understudied and provides little evidence of actual capacity or effective strategies [24]. This is alarming given the stakes that even small pilot projects have shown dramatic gains in safety when interventions are tested [2, 10, 16, 49, 51]. Thus, the real issue is not that we lack potential solutions, but that so few health facilities learn these lessons and scale up effective interventions once proven.

Overall, the findings have clear implications for health systems and policy. First, governments and health authorities must recognize that HDP is not optional but essential for any safe health system [71]. Policy-makers should prioritize investment in healthcare infrastructure and governance, ensuring that hospitals at all levels have strong physical structure, reliable critical systems, and access to modern medical supplies [24]. In fact, HDP must be integrated into health system strengthening efforts, rather than treated as a peripheral issue. Second, workforce development is important. Planners should mandate regular training, drills, and education for all hospital staff, integrated into routine accreditation and licensure processes [15, 42, 43, 54, 56, 60]. Finally, the review highlights directions for future research. There is a pressing need for multicenter and multinational studies that apply standardized metrics so that progress can be tracked objectively. Comparative studies (public versus private hospitals, urban versus rural facilities) would illuminate equity issues [24]. Interventional research, for example, implementation of specific training programs, communication upgrades, or structural retrofitting, should be a priority.

### Strengths and limitations

This review follows the scoping review methodology and hence adheres to PRISMA-ScR guidelines on robustness and transparency. Furthermore, a comprehensive search strategy across three major databases was employed to enhance inclusivity and reduce the risk of overlooking relevant studies. In addition, two independent reviewers were involved during the screening and data extraction processes to minimize personal bias. Yet, this study is not without limitations. Despite taking a wide search string and considering multiple databases, this study exclusively included only peer-reviewed quantitative English literature data, hence excluding qualitative studies, gray literature, and non-English studies, which may have led to selection bias. Second, the findings of the present study may not be generalized due to the fact that the level of HDP may vary regionally or based on contextual factors. Therefore, generalizations to other healthcare settings should be made with caution. Lastly, the recommendations outlined in the study are necessarily subjective, as informed by the interpretation of the identified gaps, and might therefore be strengthened by the addition of expert perspectives.

## Conclusions

This review demonstrates that global HDP studies have reached a saturation point in descriptive assessments but continues to neglect other essential areas, particularly structural and nonstructural resilience, equity in research representation, and standardized evaluation. Unless these systemic gaps are addressed, hospitals will remain functionally prepared but structurally vulnerable. Policymakers, health system planners, and global health actors must shift from repetitive assessments toward investments in resilient infrastructure, unified preparedness metrics, and capacity-building in underrepresented regions. By reframing preparedness from a checklist activity to a systems-level resilience agenda, this review provides a roadmap for advancing HDP research and practice worldwide.

## Supplementary Information

Below is the link to the electronic supplementary material.

Supplementary Material 1

Supplementary Material 2

## Data Availability

All data generated or analyzed during this study are included in this published article.

## Abbreviations

HDP: Hospital Disaster Preparedness
HSI: Hospital Safety Index
LMIC: Low and Middle Income Country
PRISMA-ScR: Preferred Reporting Items for Systematic Reviews and Meta-Analysis Extension for Scoping Reviews (PRISMA-ScR)
SSA: Sub-Saharan Africa
WHO: World Health Organization
