# Supplementary material for: Learning from the past: a scoping review of hospital disaster preparedness assessment
Source: BMC Emerg Med. 2026 Jan 9;26:43. doi: 10.1186/s12873-026-01474-2 (PMC12882421; doi:10.1186/s12873-026-01474-2)
Supplement: Supplementary file 1 — Supplementary Material 1 [file 12873_2026_1474_MOESM1_ESM.docx]

**Appendix A. Search strategy**

| **Search strategy area** | **Details** |
| --- | --- |
| **Geographic limit** | No limitations |
| **Language** | English |
| **Time frame** | 1 January 2015 to 31 December 2024 |
| **Databases** | PubMed, Scopus, and Web of Science |
| **Exclusion** | Qualitative studies, reviews, books, editorials, letters to editors, commentaries, conference abstracts, perspectives, and grey literature |
| **Search term** | (((((((((((“Hospital*”[Title/Abstract]) OR (“Health Facilit*”[Title/Abstract])) OR (“Healthcare Facilit*”[Title/Abstract])) AND (“Disaster Preparedness”[Title/Abstract])) OR (“Emergency Preparedness”[Title/Abstract])) OR (“Hospital Preparedness”[Title/Abstract])) OR (“Hospital Resilience”[Title/Abstract])) OR (“Hospital Safety Index”[Title/Abstract])) OR (“Structural Preparedness”[Title/Abstract])) OR (“Structural Safety”[Title/Abstract])) OR (“Nonstructural Preparedness”[Title/Abstract])) OR (“Nonstructural Safety”[Title/Abstract]) NOT (“Review*”[Title/Abstract]) |
